# Electronic Resources in Medical Libraries Issues and Solutions

**DOI:** 10.29173/jchla29607

**Published:** 2022-04-01

**Authors:** Angélique Roy

**Affiliations:** Health Sciences Librarian Queen’s University, Kingston, ON, Canada

Elizabeth Connor, M. Sandra Wood, editors. **Electronic Resources in Medical Libraries Issues and Solutions**. London: Routledge; 2021. eBook: 154 p. ISBN: 9780203051948. Price: USD$43.96. Available from: https://www.routledge.com/Electronic-Resources-in-Medical-Libraries-Issues-and-Solutions/Connor/p/book/9780203051948

Everything has changed, but nothing has changed. While this notion may conjure up sentiments expressly felt throughout the COVID-19 pandemic, it can also be applied to the rapid technological developments of the past two decades. As library professionals, it can be just as interesting to ruminate on what has changed with the proliferation of electronic resources as it is to consider what has not. While the specifics have varied – countless collections have now been migrated to digital catalogues and repositories and users now think nothing of accessing library collections from anywhere in the world – issues around the Big Deal, Open Access, interlibrary loan, website usability, and usage statistics remain a fundamental part of library work today.

For that reason, *Electronic Resources in Medical Libraries: Issues and Solutions* may be considered a foundational work for medical librarians and technicians in understanding the history of collections within and beyond their institutions. First published in 2007, this book has subsequently been re-released on two separate occasions with no changes to content. It was re-released in 2012 in print and again in 2021 in an eBook format. While the articles in this collection have not been updated in the past fourteen years – certainly a significant period of time – what is noteworthy is that many remain relevant. At the very least, they help to paint a picture of the history of electronic resource collection and management in the medical and health sciences library profession. The first article in this book, “Introduction”, was written by co-editors Elizabeth Connor and M. Sandra Wood, and was published simultaneously in Volume 4, Number 1/2 of the *Journal of Electronic Resources in Medical Libraries* which released its first issue in 2004. Connor and Wood both also served as co-editors for the journal at the time of publication and already had rich careers as widely published medical librarians. In searching for a more recent monograph, those interested in this topic might wish to read *Health Sciences Collection Management for the Twenty-First Century* edited by Susan K. Kendall which contains ten chapters written by contributors across the United States [1]. Another collection of articles edited by M. Sandra Wood, *Health Sciences Librarianship*, includes two sections dedicated to collection services and user services respectively [2].

This book contains ten papers that explore the challenges and opportunities of the ongoing digital transformation including case studies of tools and services. These focus predominantly on libraries in the United States, but also includes a case study from Qatar and an article on the Virtual Health Library’s provision of health information in Latin America and the Caribbean. Each article is a succinct representation of a topic or institutional experience and were evidently selected to represent a variety of issues across the scholarly resource landscape at the time.

The most compelling articles are those that discuss the Big Deal, Open Access, and COUNTER journal usage statistics as these remain pervasive themes for special and academic libraries. Kim and Koehler’s “Moving the Big Deal” comments that this model became fundamental for libraries embracing electronic content, but discounted journal bundles came with another cost. Oftentimes, libraries would find themselves paying for titles they did not want and otherwise would not license, and more importantly, library budgets could not always accommodate this standard. Even in 2007, the discussion centres on consortial arrangements for cost-sharing, university libraries opting out of the Big Deal, and the priority of collections being made available electronically wherever possible. While we might now wish to read about the Transformative Agreement as a direct extension of Open Access or about discipline-specific mobile apps for on-demand information, we need only look to the many library blogs and journals (many of which are Open Access) to review these current issues and trends.

As expected, there is content that is less relevant in 2022, but the overall themes represented in this book continue to be discussed albeit through a different lens. Articles such as “Integrating E-Resources into an Online Catalog: The Hospital Library Experience” is one example that shows its age with the language used, but also represents a snapshot in time. In it, the author describes the need to merge all electronic content into an Online Public Access Catalog (OPAC) using the vendor CyberTools so that staff could access this from their desktop computers and where “hyperlinks could be embedded…[to] allow users to link directly to the source”[3]. Other articles such as one exploring the legal context of a medical library’s resource offerings and another about consolidating electronic journal access using the e-journal management system TDNet can also be read for what they tell us about a point in time. The language and the tools have changed, but if we look at the table of contents for an e-resource management journal today we will still see articles on shifting to electronic access during the global pandemic, library website design, and resource accessibility across developing countries.

The target audience for this compilation is librarians and library technicians working in specialized medical libraries, but the articles are approachable for a variety of specialities seeking solutions to challenges of a similar nature. The writing of each article is clear and concise and while there is terminology specific to libraries, there is no extensive use of library jargon.

As an early career librarian working in an academic health sciences library, I was surprised by the relevance of what I learned from this book on a subject that is continuously changing. In reading “Scholarly E-Journal Pricing Models and Open Access Publishing” I read about preprint servers like Arxiv (then described as e-print) and the financial limitations of journal packages in ways that I could easily identify them as being written in the early 2000s. Yet, I could also clearly see the connections with what I am learning in my own exploration of collections and publishing. In this article, the authors discuss the challenge faced by faculty in publishing in high impact journals or paying to have their research made more widely available. Today, subscription prices continue to rise while Article Processing Charges (APC) remain a barrier for researchers at institutions without the funds to support this model of Open Access. Reading this book has also sparked my curiosity in our users’ information seeking patterns and a desire to compile and analyze COUNTER usage reports and ILS metrics for eBooks and journals. The contents of at least a handful of articles struck me as being very similar to the discussions we are having daily in the pursuit of open scholarship and increased discoverability of information resources.

I would recommend this book to a fellow early career librarian or technician with an elementary understanding of e-resource management in the health sciences, especially for those seeking context for how their library came to operate as it currently does. It may not be of relevance to those who have witnessed these developments firsthand throughout their careers. Nevertheless, as I have experienced, it may serve as a springboard for discussions about the library’s role in enacting meaningful change: to continue to seek solutions for reducing the financial burden of authors and their institutions in providing access to electronic materials and in tailoring our collections to the needs of our users.
